# Bovine Tuberculosis as a Neglected Zoonotic Disease in Mexico and Latin America: Epidemiological Challenges, Diagnostic Insights, and Public Health Implications in Emerging Economies

**DOI:** 10.3390/vetsci13030259

**Published:** 2026-03-11

**Authors:** Luis M. Rodríguez-Martínez, Jose L. Chavelas-Reyes, Carlo F. Medina-Ramírez, Jorge A. Valdés-González, Eli Fuentes-Chávez, Carlos A. Ríos-Saldaña, Miguel A. de León-Zapata, Josefina G. Rodríguez-González

**Affiliations:** 1 Centro de Estudios e Investigaciones Interdisciplinarios, Universidad Autónoma de Coahuila, Saltillo 25280, Mexico; 2 Centro de Biotecnología Genómica, Instituto Politécnico Nacional, Reynosa 88710, Mexico

**Keywords:** *Mycobacterium bovis*, bovine tuberculosis, interdisciplinary, zoonotic disease

## Abstract

Bovine tuberculosis (bTB), caused by *Mycobacterium bovis*, remains one of the most relevant zoonotic diseases worldwide due to its dual impact on livestock production and human health. Although zoonotic tuberculosis has been virtually eradicated from cattle in a few settings, particularly in Australia, the disease persists in much of Latin America, Africa, and Asia, where it continues to limit cattle productivity and pose a threat to public health through the consumption of unpasteurized dairy products and occupational exposure. This review integrates historical, epidemiological, clinical, and molecular perspectives of bTB, with particular emphasis on Mexico, highlighting the role of wildlife reservoirs, socioeconomic factors, and diagnostic limitations in maintaining endemicity. Recent advances in molecular epidemiology, such as PCR, MIRU-VNTR, and whole-genome sequencing, provide promising avenues for surveillance and control. Finally, we discuss the importance of adopting a One Health framework that bridges veterinary, medical, and environmental approaches to achieve sustainable control of this silent zoonosis.

## Simple Summary

Bovine tuberculosis is a contagious disease of cattle that can also infect people, mainly through close contact with sick animals or by consuming raw milk and fresh cheeses made from infected cows. Its incidence has been reduced in several rich countries, but in Mexico and other Latin American nations it remains a major problem in dairy regions and rural communities, where veterinary services, food controls, and milk pasteurization are often limited. This article reviews historical background, current distribution, symptoms in animals and humans, available tests, and new genetic tools to track the bacteria. It explains that the disease persists because common tests can fail to detect infected animals and because animal health, human health, and food safety sectors rarely coordinate their actions. One Health is analyzed as an integrative epidemiological framework that links surveillance and control actions in animals, humans, and the environment, using shared data to identify high-risk areas, transmission routes, and vulnerable populations. By coordinating veterinarians, physicians, and food authorities, this approach strengthens early detection, the tracing of infection sources, and targeted interventions to reduce bovine tuberculosis in farmers, consumers, and rural communities.

## 1. Introduction

Bovine tuberculosis (bTB), caused by *Mycobacterium bovis*, remains one of the most persistent and underestimated zoonotic diseases worldwide, particularly in low- and middle-income regions where animal health surveillance, food safety regulations, and intersectoral coordination remain poor. Despite sustained eradication efforts in several high-income countries, bTB continues to circulate endemically in large parts of Latin America, Africa, and Asia, generating a dual burden: substantial economic losses in livestock production systems and a continuous, often underdiagnosed, threat to human health. This persistent circulation highlights not only biological complexity but also structural weaknesses in surveillance, diagnostics, and policy integration [1]. In contrast, several Western European countries, Canada, and parts of the United States have achieved freedom from or a very low prevalence of bovine tuberculosis in cattle through long-standing test-and-slaughter programs and strict movement controls, highlighting the gap between these settings and endemic regions such as Mexico and other Latin American countries [2,3].

From a global perspective, *M. bovis* represents a unique epidemiological challenge within the Mycobacterium Tuberculosis Complex (MTBC) due to its broad host range and its ability to persist across interconnected domestic, wildlife, and human populations. While human tuberculosis is predominantly attributed to *M. tuberculosis*, zoonotic tuberculosis caused by *M. bovis* is frequently overlooked, in part because routine clinical diagnostics in humans rarely differentiate species within the complex. As a result, the true burden of zoonotic tuberculosis remains underestimated, particularly in regions where exposure to livestock and the consumption of unpasteurized dairy products are common practices [4].

In Latin America, and especially in Mexico, bTB exhibits marked heterogeneity across production systems and regions. Dairy herds consistently show higher apparent prevalence based on tuberculin testing than beef cattle, with herd-level values reaching up to 16% in certain endemic areas [5], while beef systems often remain below 1%. However, localized animal-level prevalences exceeding 25–35% have been documented in outbreak investigations, underscoring the limitations of aggregated national statistics and revealing the existence of micro-epidemiological hotspots [6,7,8].

Importantly, the persistence of bTB in Mexico occurs despite decades of control efforts, including systematic tuberculin testing, the culling of reactor animals, and differentiated strategies for beef and dairy sectors [9]. This apparent paradox points to critical limitations of conventional surveillance tools, particularly their reduced sensitivity in early or latent infections and their inability to fully capture transmission dynamics at the animal–human–environment interface. In this context, wildlife reservoirs, informal dairy markets, and socio-economic constraints play a central role in sustaining endemic transmission cycles [9,10].

Since the early 2000s, molecular epidemiology has transformed the understanding of tuberculosis transmission. Techniques such as PCR-based detection, spoligotyping, MIRU-VNTR, and, more recently, whole-genome sequencing (WGS) have provided unprecedented resolution for tracing transmission pathways, identifying dominant genotypes, and establishing direct links between animal and human cases. In Mexico, molecular studies have revealed overlapping *M. bovis* genotypes in cattle, dairy products, and human patients, providing compelling evidence of ongoing zoonotic transmission and exposing the limitations of compartmentalized surveillance system [11].

Nevertheless, despite the availability of these advanced tools, their integration into routine control programs remains limited. Molecular diagnostics are often confined to research settings, while national eradication strategies continue to rely heavily on traditional immunological tests. This gap between technological capability and operational implementation represents one of the main barriers to effective bTB control in endemic regions [12,13].

Within this framework, the One Health approach has been widely promoted as a conceptual solution to zoonotic diseases such as bTB. However, in practice, its implementation has been uneven, frequently remaining at the level of policy discourse rather than operational integration [14]. The lack of systematic data sharing between veterinary and public health sectors, limited molecular differentiation of clinical isolates, and insufficient consideration of environmental and wildlife reservoirs continue to undermine coordinated responses [15,16].

This review critically examines bovine tuberculosis as a zoonotic disease in Mexico and Latin America, moving beyond descriptive summaries to provide an integrated analysis of epidemiological patterns, diagnostic strategies, and molecular surveillance tools. By synthesizing available evidence and highlighting key gaps in current knowledge and practice, we aim to identify strategic priorities for future research and control efforts. In doing so, this article positions molecular epidemiology and One Health integration not as optional enhancements, but as essential components for the sustainable control and eventual elimination of this neglected zoonosis.

## 2. Narrative Review Methods

This narrative review was designed to synthesize epidemiological, clinical, and molecular evidence on bovine and zoonotic tuberculosis with an emphasis on Mexico and Latin America. We conducted comprehensive searches in PubMed, Scopus, Web of Science, Google Scholar, and the MDPI Open Access database, covering publications from approximately 2000 up to early 2025. Search terms included combinations of “Mycobacterium bovis”, “bovine tuberculosis”, “zoonotic tuberculosis”, “Mexico”, “Latin America”, “spoligotyping”, “MIRU-VNTR”, “whole-genome sequencing”, “molecular epidemiology”, “One Health”, and “control program”. No formal language restrictions were applied at the search stage, but we focused on peer-reviewed and indexed publications in English and Spanish. Reference lists of key articles and relevant international reports (WHO, WOAH, FAO) were manually screened to identify additional records. The objective was not to conduct a systematic review in the PRISMA sense, but to integrate converging lines of evidence and highlight knowledge gaps and operational challenges relevant to bovine tuberculosis as a neglected zoonotic disease in emerging economies. During manuscript revision, AI-assisted tools were used exclusively for language refinement; they were not employed to generate original data, analyses, or interpretations.

## 3. Tuberculosis as a Zoonosis

Tuberculosis is deeply intertwined with the development of microbiology and modern infectious disease control. In 1882, Robert Koch announced the discovery of the tubercle bacillus, consistently identifying *M. tuberculosis* in affected tissues and establishing the microbial etiology of the disease. This milestone was pivotal for bacteriology, as it provided clear evidence of the infectious nature of tuberculosis and laid the foundations for systematic public health interventions. The immediate impact of Koch’s discovery was reflected in the creation of dedicated institutions such as the National Tuberculosis Association in the United States in 1904, while one year later, Koch was awarded the Nobel Prize in Medicine for his contributions [17].

Subsequent advances refined the understanding of tuberculosis. In 1898, Theobald Smith demonstrated the existence of differences between the bacillus infecting humans and the one affecting cattle, confirming that *M. bovis* was responsible for bovine tuberculosis. This differentiation consolidated the recognition of *M. bovis* as a zoonotic pathogen capable of infecting humans and animals alike. At the beginning of the 20th century, tuberculosis was among the leading causes of mortality in humans and domestic animals. In response, control measures were progressively implemented in developed countries, including the introduction of the tuberculin skin test by Charles Mantoux, the culling of infected animals, and sanitary measures such as milk pasteurization, which drastically reduced transmission through dairy products [18,19].

According to recent global estimates, approximately 10 million people develop active tuberculosis each year, corresponding to an incidence of roughly 130–140 cases per 100,000 population, with the highest burden concentrated in low- and middle-income countries. Within this overall landscape, Latin American countries generally show intermediate incidence levels compared with high-burden regions in Africa and Asia, but substantial subnational heterogeneity persists, particularly in rural and marginalized populations where close contact with livestock and consumption of unpasteurized dairy products are more frequent. This context underscores the importance of understanding bovine tuberculosis not only as an animal health problem but also as a contributor to the broader human tuberculosis burden in endemic settings [20].

The term “tuberculosis” derives from the characteristic tubercles, or granulomatous nodules, formed in affected tissues. Historical records indicate that, before modern interventions, human cases were often associated with the consumption of unpasteurized milk or close contact with infected cattle. Over time, bovine tuberculosis was recognized as a notifiable disease by the World Organization for Animal Health (WOAH), underscoring its dual impact: as a cause of losses in animal productivity and as a zoonotic risk restricting trade and food safety. Even today, *M. bovis* is still reported in 44% of surveyed territories worldwide, where it persists in both livestock and wildlife reservoirs [21].

The control and eradication of bTB have historically required extensive financial, logistical, and technical resources. For instance, Spain initiated a national campaign in 1965, which was later co-financed by the European Union from 1990 onwards [22]. The strategies applied included systematic intradermal tuberculin testing with bovine purified protein derivative (PPD), the culling of reactor animals, and follow-up through bacteriological and histopathological confirmation. Similar campaigns in other European countries gradually reduced prevalence, but complete eradication has proven elusive in areas where wildlife maintains transmission cycles [23].

In Latin America, different approaches have been attempted with variable success. Reports of tuberculosis in calves in several countries underscored that infection could occur at very early stages of life, a finding that was initially unexpected and highlighted the need for timely intervention to prevent the establishment of infectious reservoirs within herds. This recognition led to proposals for complementary measures, including vaccination strategies aimed at reducing incidence and improving immune responses. Although recombinant vaccines have been explored, their use remains limited due to concerns about interference with diagnostic tests and the need for long-term validation [24].

Mexico has played a particularly active role in the regional fight against bovine tuberculosis. Since the 1970s, the coordinated establishment of the Programa Nacional de Biológicos Veterinarios (PRONABIVE) has enabled the large-scale production of antigens and diagnostic reagents, which supported the implementation of the National Campaign against Bovine Tuberculosis coordinated by the Servicio Nacional de Sanidad, Inocuidad y Calidad Agroalimentaria (SENASICA).These programs included differentiated strategies for beef and dairy herds, reflecting the uneven distribution of the disease across production systems. Nevertheless, regions of high livestock density, especially those with dairy predominance, continue to exhibit persistent infection despite decades of intervention [25].

From a broader historical perspective, tuberculosis illustrates the complexities of zoonoses. Transmission from animals to humans occurs through aerosols and contaminated dairy products, while human patients with active pulmonary TB contribute to the circulation of the pathogen via respiratory droplets (Pflügge droplets), which remain suspended in the air and facilitate contagion. This bidirectional dynamic between species reinforces the concept of tuberculosis as a shared disease requiring coordinated control strategies [26].

## 4. Molecular Epidemiology

Molecular epidemiology has become a cornerstone for understanding the persistence, transmission dynamics, and zoonotic impact of bovine tuberculosis (bTB) caused by *Mycobacterium bovis*. Unlike classical epidemiological approaches based on prevalence estimates and clinical surveillance, molecular tools provide strain-level resolution, enabling the reconstruction of transmission pathways across animal populations, wildlife reservoirs, and humans [27]. This level of resolution is particularly critical in endemic settings such as Mexico and other Latin American countries, where heterogeneous production systems and fragmented surveillance obscure transmission dynamics [24].

In Mexico, molecular studies have consistently revealed a complex and non-random distribution of *M. bovis* genotypes. Early spoligotyping surveys identified dominant genotypes circulating in high-density dairy regions, alongside geographically restricted variants in northern and central states. This spatial structuring suggests that bTB transmission is shaped not only by animal movement but also by regional production practices, biosecurity levels, and ecological interfaces with wildlife. Importantly, these findings challenge the assumption of homogeneous national transmission patterns and highlight the existence of localized transmission networks that sustain endemicity [28].

One of the most significant contributions of molecular epidemiology has been the demonstration of overlapping *M. bovis* genotypes between cattle and humans within the same regions [29]. The identification of identical or closely related spoligotypes in livestock, dairy products, and human clinical isolates provides robust evidence of ongoing zoonotic transmission. This data strongly suggest that a substantial fraction of human tuberculosis cases attributed generically to the *M. tuberculosis* complex may, in fact, be of bovine origin, particularly in regions where unpasteurized dairy consumption and occupational exposure remain common [30].

The application of MIRU-VNTR typing has further refined this picture by increasing discriminatory power beyond spoligotyping. By resolving strain-level variation within dominant genotypes, MIRU-VNTR analyses have revealed both clonal expansion within dairy systems and potential cross-transmission between dairy and beef herds. This overlap complicates epidemiological interpretation, as it remains unclear whether beef cattle infections arise from independent transmission cycles or from spillover events linked to dairy production environments. Such uncertainty underscores the limitations of conventional surveillance systems that lack traceability and molecular resolution [31]. Despite its higher discriminatory power compared to spoligotyping, MIRU-VNTR is not free from interpretative limitations. Homoplasy at certain loci can lead to convergent profiles that do not reflect true recent common ancestry, potentially obscuring or misrepresenting transmission links in endemic settings. Therefore, MIRU-VNTR data should be interpreted cautiously and, whenever possible, complemented with higher-resolution approaches such as whole-genome sequencing [32].

More recently, whole-genome sequencing (WGS) has emerged as the most powerful tool for molecular epidemiology of bTB. WGS enables high-resolution phylogenetic reconstruction, allowing the identification of transmission clusters based on single-nucleotide polymorphism (SNP) differences. In Mexico, WGS-based studies have demonstrated close genetic relationships between isolates from cattle, artisanal cheese, and humans, with SNP distances consistent with recent transmission events. These findings provide definitive evidence of foodborne and occupational zoonotic pathways and reveal the inadequacy of surveillance strategies that treat animal and human tuberculosis as separate entities [33].

Beyond confirming zoonotic links, WGS has reshaped the understanding of *M. bovis* population structure [34]. The identification of regional clades and subclades may reflect long-term adaptation of strains to specific ecological and production contexts. This genomic structuring has direct implications for control strategies, as it suggests that uniform national interventions may be less effective than regionally tailored approaches informed by molecular data (Table 1). Furthermore, the low overall genetic diversity of *M. bovis*, coupled with signals of clonal expansion, supports the hypothesis that ongoing transmission is driven by persistent reservoirs rather than frequent introductions of novel strains [35,36].

It is important to acknowledge that the available molecular evidence from Latin America is not evenly distributed across the region: most published datasets originate from a limited number of high-burden areas in Mexico, such as Baja California, Hidalgo, Querétaro and other central dairy states, as well as from a few additional countries, which may introduce publication bias and limit the generalizability of current conclusions [13,28].

Despite these advances, the integration of molecular epidemiology into routine control programs remains limited in most endemic countries. In Mexico, molecular tools are largely confined to research projects and retrospective analyses, rather than being systematically incorporated into surveillance and decision-making. As a result, critical opportunities to identify transmission hotspots, trace infection sources, and evaluate the effectiveness of control measures are frequently missed. From a public health perspective, this disconnect has significant consequences [37]. The lack of routine molecular differentiation between *M. bovis* and *M. tuberculosis* in human cases perpetuates underestimation of zoonotic tuberculosis and obscures its true epidemiological impact. Without molecular data linking animal and human infections, control programs remain reactive rather than preventive, focusing on late-stage detection rather than interruption of transmission chain [38].

## 5. Transmission and Spread

**Figure 1:**
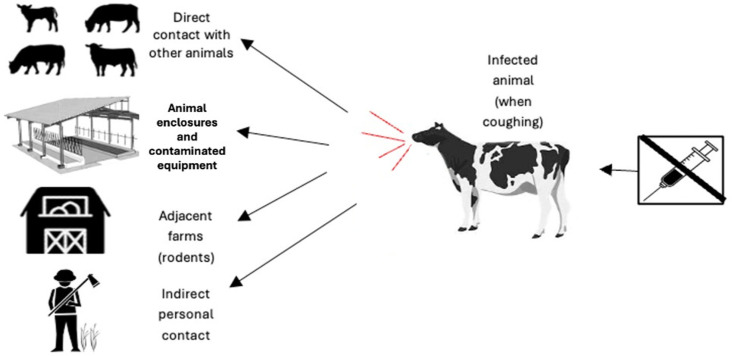
Conceptual overview of major transmission pathways of *Mycobacterium bovis* into cattle herds; arrows indicate the direction of possible transmission towards cattle.

Zoonotic transmission of bovine tuberculosis occurs primarily through direct or indirect contact with infected domestic or wild animals. The respiratory route predominates, whereby infected cattle generate aerosols laden with bacilli during coughing, sneezing, or exhalation, which susceptible animals inhale in proximity. Figure 1 depicts the principal pathogen entry routes into livestock herds; the struck-through syringe denotes the absence of approved preventive vaccination, rendering animals vulnerable via direct contact with infected cattle, contaminated fomites, rodents, or other farm species, as well as indirect transmission involving humans [39].

Another significant transmission pathway is the digestive route, especially relevant in calves and humans consuming raw milk or unpasteurized dairy products from infected cows. In cattle, infection can also occur through ingestion of saliva, nasal secretions, or contaminated materials in feeders and troughs, where even a single infected animal may act as a silent disseminator before showing clinical signs [40].

Other less frequent routes have been documented, including intrauterine transmission, venereal spread, or infection through bite wounds. Indirect transmission via contaminated food, water, or pastures is also common in extensive production systems and among wildlife populations [41,42]. These environmental pathways explain the persistence of the disease in ecosystems, as the bacterium can remain viable outside the host and perpetuate hidden transmission chains [43].

Cattle constitute the primary reservoir of *M. bovis*, but the persistence of the pathogen in multispecies communities has been widely demonstrated, complicating the eradication efforts. Among the most studied wild reservoirs are Eurasian badgers, deer, and wild boar in Europe [23]; brushtail possums and ferrets in New Zealand [44]; white-tailed deer and bison in North America [45]; African buffalo in South Africa [46]; and water buffalo in Australia [47]. In these contexts, sanitary control becomes highly challenging, since the bacteria circulate between domestic and wild hosts, maintaining infection chains that are difficult to interrupt. Furthermore, several carnivores and scavengers, such as coyotes, wolves, lions, lynxes, and bears, can also acquire infection through contact with contaminated carcasses. Species such as wild boars are particularly susceptible to environmental exposure and are now recognized as important epidemiological reservoirs [48]. In contrast, long-term integrated programs in countries such as New Zealand, which combine intensive testing in cattle with strict movement control and sustained management of brushtail possum populations, illustrate that coordinated multi-host strategies can markedly reduce bovine tuberculosis prevalence [49].

The introduction of bovine tuberculosis into previously uninfected herds occurs predominantly through the movement of subclinically infected cattle, either via trade, restocking, or communal grazing systems. At this herd level, asymptomatic animals in latent stages of infection can silently transport the pathogen to new locations, triggering outbreaks that may remain undetected for prolonged periods. These dynamics highlight that cattle movements and contact networks between farms are critical drivers of regional spread [50].

Once *M. bovis* has entered a herd, transmission at the animal level is primarily driven by close respiratory contact and, to a lesser extent, by ingestion of contaminated milk, colostrum, or environmental materials such as feed and water. Infected cattle can shed bacilli in respiratory secretions, saliva, and, in some cases, milk, exposing both herd mates and young calves before clinical signs become apparent [51]. Distinguishing between herd-level introduction through animal movements and within-herd transmission mechanisms is essential for designing effective control strategies, as measures targeting trade and traceability differ from those required to reduce animal-to-animal spread within affected herds [52].

In humans, transmission occurs mainly via respiratory and digestive routes. Populations most exposed are those with close and prolonged contact with livestock. Clinical lesions can take years to manifest, as the incubation period is highly variable, ranging from a few months to over a decade. This latency complicates diagnosis and facilitates the silent dissemination of the pathogen [53].

The WHO estimates that *M. bovis* is responsible for a considerable proportion of extrapulmonary tuberculosis cases, particularly in countries with high prevalence of bovine tuberculosis and without universal access to pasteurization. Although zoonotic tuberculosis cases are usually estimated at 1–2% of total human tuberculosis cases, this figure is believed to be underestimated due to the absence of routine differentiation between *M. bovis* and *M. tuberculosis* in many public health systems [54].

Zoonotic transmission has been described in rural and indigenous populations of Latin America, Africa, and Asia, especially linked to the consumption of raw milk, artisanal cheeses, and direct contact with infected animals. In Mexico, several studies have confirmed isolations of *M. bovis* from human cases, with significant prevalence in livestock-producing regions where veterinary surveillance is limited [55]. Globally, it is estimated that 94% of the human population resides in countries where effective bovine tuberculosis control programs are not implemented, and in these contexts, *M. bovis* can account for up to 10% of tuberculosis cases [56].

Unlike *M. tuberculosis*, specific treatment considerations will be required, as discussed below [57]. This resistance complicates therapeutic schemes and necessitates adjusted protocols, especially in health systems where bacterial differentiation is not routinely performed. The absence of differential diagnosis can result in ineffective treatments, longer disease duration, and greater risk of dissemination [58]. In contrast, in industrialized countries, the combination of systematic eradication campaigns in cattle, milk pasteurization, and strict biosecurity practices has markedly reduced the incidence of zoonotic tuberculosis, demonstrating the effectiveness of coordinated veterinary and public health measures [59].

Taken together, these transmission pathways underscore the limitations of livestock-centered control strategies that fail to account for wildlife reservoirs, foodborne exposure, and human–animal interfaces. This complexity reinforces the need for integrated surveillance frameworks informed by molecular epidemiology to effectively interrupt hidden transmission chains [60,61].

Molecular epidemiology studies from high-income settings have further illustrated these multi-level dynamics. In the United States, whole-genome sequencing combined with field investigations has documented MTBC transmission between humans and cattle, highlighting the bidirectional nature of spillover at the human–livestock interface [62]. In the United Kingdom, analyses of cattle and badger isolates have demonstrated complex multi-species transmission networks in endemic regions, underscoring the importance of wildlife reservoirs and animal movements in sustaining bovine tuberculosis [63,64].

## 6. Clinical Manifestations of Bovine Tuberculosis in Animals and Humans

The bTB, driven by *M. bovis* infection, follows a slow and often silent progression, with the capacity to persist latently within the host for long periods. This latency allows apparently healthy animals to function as silent carriers and disseminators of infection within herds [65,66]. The progression of the disease in cattle is highly variable: while some animals develop symptoms shortly after infection, in most cases the disease advances slowly, with no evident manifestations for months or even years [67].

When present, clinical signs in cattle are usually nonspecific and include progressive weight loss, weakness, anorexia, dyspnea, intermittent fever, lymphadenomegaly, and chronic cough [68]. Tuberculous lesions can develop in different organs, predominantly in the lungs and retropharyngeal, bronchial, mesenteric, or mediastinal lymph nodes. Depending on the site of infection, affected cattle may show respiratory signs such as chronic wet cough or digestive symptoms, depending on the organs involved. In the terminal stages, advanced deterioration is evident, with severe weight loss, persistent fever, and acute respiratory compromise. However, due to the nonspecificity of symptoms, clinical diagnosis of bTB is of limited value and must always be confirmed by intradermal, histopathological, or molecular testing [69,70].

In humans, cases of tuberculosis caused by *M. bovis* represent a smaller proportion compared to those produced by *M. tuberculosis*, but they are of great relevance in zoonotic contexts. From a clinical perspective, the most affected groups are agricultural workers, veterinarians, slaughterhouse personnel, and consumers of unpasteurized dairy products, who often develop extrapulmonary forms of the disease. Clinical manifestations are often similar to those of conventional tuberculosis, but with a higher tendency toward extrapulmonary forms. Reported symptoms include intermittent cough, fluctuating fever, weakness, loss of appetite, weight loss, diarrhea, and lymphadenopathy, as well as low-grade pneumonia. In advanced cases, granulomatous lesions may appear in lymph nodes, the gastrointestinal tract, the liver, and other organs [71].

A relevant clinical aspect is that *M. bovis* infections require modified treatment regimens, since the bacterium is intrinsically resistant to pyrazinamide. This resistance has important implications for treatment since therapeutic schemes must be adjusted, especially in countries where human cases are not routinely differentiated by bacterial species. Failure to make this distinction can lead to inappropriate treatment, prolonged disease courses, and greater potential for dissemination [72,73].

Although the clinical picture of *M. bovis* and *M. tuberculosis* infections is often indistinguishable, studies indicate a higher frequency of extrapulmonary tuberculosis in *M. bovis* cases, especially in lymph nodes and the gastrointestinal tract. This pattern is consistent with the zoonotic nature of infection, frequently associated with ingestion of contaminated dairy products, and highlights the importance of considering *M. bovis* as a potential etiological agent in human patients with extrapulmonary tuberculosis [74].

## 7. Diagnosis

Bovine tuberculosis (bTB) remains a major diagnostic challenge due to its chronic course and the heterogeneous performance of available detection tools across different stages of infection, a limitation that is particularly evident in endemic regions such as Mexico and other Latin American countries [24]. Constraints in sensitivity and specificity directly affect early case identification, weaken eradication programs, and increase the risk of zoonotic transmission, especially where veterinary infrastructure and laboratory capacity are limited [75]. Despite decades of methodological development and program implementation, no single diagnostic approach provides sufficient accuracy across all infection stages and epidemiological contexts, and so national control programs increasingly incorporate complementary immunological, serological, and molecular tools, each with distinct strengths and limitations that must be adapted to local conditions [76].

**Figure 2:**
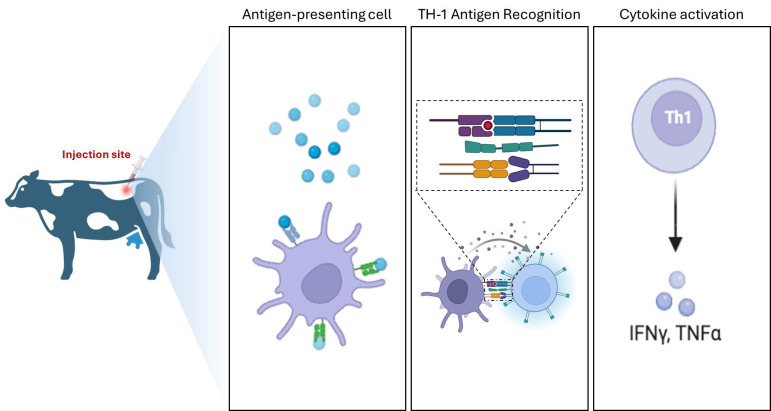
Conceptual representation of the Th1-mediated immune response underlying cellular immunity–based diagnostic assays for bovine tuberculosis. Antigen presentation and cytokine-driven inflammation constitute the biological basis of delayed-type hypersensitivity responses exploited by tuberculin-based surveillance tools [78]. The red arrow denotes the intradermal injection site, dashed arrows indicate antigen recognition and T cell activation, and the solid black arrow indicates cytokine release (IFNγ, TNFα).

Most national programs continue to implement a screening-and-culling strategy in which the intradermal tuberculin skin test serves as the primary tool for large-scale surveillance (Figure 2). While this approach enables population-level monitoring, its diagnostic performance is inherently variable. False-positive reactions associated with exposure to environmental mycobacteria and false-negative results in animals at early or advanced stages of infection remain well-recognized limitations. Consequently, tuberculin testing is increasingly integrated with ancillary diagnostic tools, including serological assays, molecular detection methods, and confirmatory culture techniques, to improve overall detection accuracy [77].

From a One Health perspective, the accurate diagnosis of bovine tuberculosis extends well beyond the veterinary domain. In settings where consumption of unpasteurized dairy products is common or where close livestock–human contact persists, the capacity to rapidly and reliably detect *M. bovis* has direct implications for food safety and public health. The lack of routine differentiation between *M. bovis* and *M. tuberculosis* in human clinical laboratories contributes to systematic underestimation of zoonotic tuberculosis and may result in inappropriate treatment regimens, particularly given the intrinsic resistance of *M. bovis* to pyrazinamide [79].

In practice, most eradication strategies continue to depend heavily on the intradermal tuberculin skin test as a primary screening tool. While this test enables large-scale field application at relatively low cost, its performance is highly variable. Sensitivity is reduced during early infection and advanced disease, while specificity may be compromised by exposure to environmental mycobacteria. These limitations have direct epidemiological consequences, as undetected infected animals may remain within herds and sustain transmission [80].

To address these shortcomings, ancillary immunological assays such as interferon-gamma release assays (IGRAs) have been incorporated in some settings. These tests improve early detection and reduce the time required for diagnosis; however, their higher cost, need for laboratory infrastructure, and reduced specificity in certain epidemiological contexts limit widespread adoption. Serological assays, particularly ELISA-based methods, provide additional value by identifying animals with established humoral responses, but their reduced sensitivity in early infection restricts their use as standalone tools [81].

Molecular diagnostics, including PCR-based assays, have significantly enhanced the capacity for the rapid and specific detection of *M. bovis*. Unlike immunological tests, molecular methods directly detect bacterial genetic material, enabling confirmation within days rather than weeks. However, their performance depends strongly on sample quality, bacterial load, and laboratory capacity. As a result, molecular diagnostics are often applied as confirmatory tools rather than as primary screening methods [73,82].

Whole-genome sequencing (WGS), while not a frontline diagnostic tool, has redefined confirmatory diagnosis and epidemiological investigation by providing strain-level resolution and enabling linkage between animal, food, and human cases. Nevertheless, its implementation remains largely restricted to reference laboratories and research settings, limiting its immediate impact on routine disease control [13].

Taken together, these diagnostic approaches illustrate a fundamental trade-off between scalability, cost, resolution, and epidemiological value. In endemic regions, reliance on a single diagnostic modality is insufficient and may generate false confidence in disease status. Instead, integrated diagnostic algorithms that combine field-applicable screening tests with targeted molecular confirmation represent the most robust strategy for effective surveillance and control.

However, in many endemic countries, these integrated diagnostic algorithms are not widely implemented due to a combination of financial, infrastructural, and institutional barriers. Limited laboratory capacity, shortages of trained personnel, and competing priorities within veterinary services constrain the routine use of molecular confirmation, while fragmented funding mechanisms and the absence of clear incentives for producers reduce the feasibility of adopting multi-step testing protocols. National veterinary authorities, in collaboration with public health agencies and, where appropriate, producer organizations and international partners, would need to allocate dedicated resources and establish explicit policy frameworks to support the gradual integration of these algorithms into bovine tuberculosis control programs [83,84].

## 8. One Health and Public Health Implications

The persistence of bovine tuberculosis (bTB) in endemic regions underscores the limitations of sectorial approaches to disease control and highlights the need for a genuinely integrated One Health framework. Although the interconnectedness of animal, human, and environmental health is widely acknowledged at the policy level, its operational implementation in the context of bTB remains fragmented, particularly in low- and middle-income countries such as those in Latin America [85,86].

At the global level, bovine tuberculosis has been recognized as a priority transboundary animal disease by international agencies such as the World Organization for Animal Health and the Food and Agriculture Organization, which provide technical standards and guidance for diagnosis, control, and surveillance [87,88]. In some regions of Mexico, microbiological investigations of unpasteurized dairy products, particularly artisanal cheeses and raw milk, have reported MTBC rates of up to 15%, although estimates vary according to region, product type, and sampling strategy; in these studies, raw milk and cheese samples were inoculated onto Stonebrink and Lowenstein–Jensen media to obtain microbial cultures, and mycobacterial isolates were identified to the biotype level using standard biochemical tests, including niacin and nitrate assays [30,89]. Across Latin America, *M. bovis* has been estimated to account for approximately 2–8% of human tuberculosis cases, based mainly on hospital-based series and national reports that include species-level identification. However, this range probably underestimates the true burden of zoonotic tuberculosis, as routine differentiation within the MTBC remains uncommon in many public health systems [90,91].

In Mexico, veterinary surveillance of *Mycobacterium bovis* is largely conducted through livestock-focused eradication programs, while human tuberculosis surveillance is managed independently within public health systems [92]. This institutional separation has important epidemiological consequences. Human clinical laboratories rarely perform routine differentiation between *M. bovis* and *M. tuberculosis*, resulting in systematic underestimation of zoonotic tuberculosis and obscuring transmission links between animals, food products, and humans. Consequently, zoonotic cases are frequently misclassified, and treatment regimens may be suboptimal due to the intrinsic resistance of *M. bovis* to pyrazinamide. This resistance complicates therapeutic management, delays appropriate treatment when bacterial differentiation is not performed, and increases the risk of prolonged infection and transmission [34,93].

Beyond the underestimation of zoonotic tuberculosis in national statistics, the potential contribution of human-associated transmission to the overall dynamics of *M. bovis* remains poorly defined. Most surveillance systems implicitly treat human *M. bovis* infections as epidemiological dead ends, yet reports of person-to-person transmission, particularly among immuno-compromised individuals and within close-contact settings, indicate that secondary spread can occur under specific circumstances [94]. As whole-genome sequencing is progressively incorporated into tuberculosis surveillance, additional clusters involving *M. bovis* are likely to be detected, revealing transmission networks that combine animal-to-human and, occasionally, human-to-human pathways. Integrating routine species- and strain-level characterization of human tuberculosis cases into One Health surveillance would therefore improve source attribution and provide more accurate quantification of the relative contribution of each transmission route in endemic regions [95].

Food safety represents a critical interface where One Health failures become particularly evident. In regions with high consumption of unpasteurized milk and artisanal dairy products, molecular studies have demonstrated the presence of *M. bovis* strains genetically indistinguishable from those circulating in local cattle populations. These findings reveal a direct foodborne transmission pathway that is insufficiently addressed by current surveillance systems, which often lack coordination between animal health authorities and food safety regulators. Informal dairy markets, limited cold-chain infrastructure, and cultural practices further exacerbate this risk, particularly in rural and marginalized communities [96].

Wildlife reservoirs add an additional layer of complexity to the One Health challenge. In Mexico and other Latin American countries, interactions between cattle and wildlife species such as deer, wild boar, and other mammals create ecological interfaces that sustain transmission cycles beyond the reach of conventional livestock-focused interventions [97]. Despite growing molecular evidence supporting the role of wildlife in maintaining *M. bovis* circulation, wildlife surveillance remains sporadic and is rarely integrated into national tuberculosis control strategies. This gap limits the effectiveness of eradication programs and contributes to reinfection of ostensibly controlled herds [98,99].

Molecular epidemiology provides a unique opportunity to operationalize One Health principles by generating data that are inherently integrative. Whole-genome sequencing and high-resolution genotyping has demonstrated their capacity to link animal, food, and human isolates within shared transmission networks, offering actionable insights that transcend disciplinary boundaries. However, in most endemic settings, these tools remain confined to academic studies rather than being incorporated into routine surveillance and policy decision-making. This disconnect between scientific capability and institutional practice represents a critical bottleneck for One Health implementation, as advanced molecular tools remain confined to research settings instead of being integrated into routine surveillance and decision-making [100].

From a public health perspective, the failure to integrate veterinary and human health data perpetuates reactive rather than preventive strategies. Control efforts continue to focus on late-stage detection and culling, while upstream interventions—such as targeted surveillance of high-risk dairy regions, molecular monitoring of food products, and systematic differentiation of clinical isolates—are underutilized. A One Health-oriented strategy would prioritize shared databases, coordinate outbreak investigations, and harmonized diagnostic protocols across sectors [101,102].

Ultimately, effective control of bovine tuberculosis requires moving beyond rhetorical endorsement of One Health toward measurable operational integration. Strengthening intersectoral collaboration, expanding molecular surveillance capacity, and aligning veterinary, food safety, and public health policies are essential steps to reduce both animal and human disease burden. In endemic regions such as Mexico and Latin America, the incorporation of molecular epidemiology into a functional One Health framework is not merely an academic aspiration but a practical necessity for achieving sustainable control of this persistent zoonosis [103].

## 9. Recommendations for One Health Implementation

Implementing an effective One Health framework for bovine tuberculosis in Mexico and Latin America requires translating conceptual alignment into concrete operational measures across sectors. First, intersectoral collaboration between veterinary services, public health authorities, food safety regulators, and wildlife agencies should be formalized through permanent coordination mechanisms rather than ad hoc project-based initiatives. These mechanisms may include interagency committees with clearly defined mandates, shared epidemiological indicators, and regular joint outbreak investigations in high-risk dairy regions and livestock–wildlife interfaces. Structural barriers, such as fragmented information systems, unclear legal responsibilities, and limited resources, can be partly mitigated by establishing shared reporting platforms and allocating specific budgets for cross-sectoral surveillance activities [15].

Second, molecular surveillance capacity must be expanded beyond reference laboratories and academic research groups. Priority actions include the development of regional laboratories capable of routine genotyping or targeted whole-genome sequencing, the standardization of protocols for sample collection and data analysis, and the training of personnel in both veterinary and human health systems. Sustainable funding may derive from public–private partnerships with the dairy and meat industries, the integration of molecular tools into national tuberculosis programs, and the strategic use of existing laboratory networks established for other priority pathogens. Demonstrating how molecular data can directly inform decisions—such as identifying transmission hotspots, evaluating animal movement controls, or targeting high-risk value chains—may facilitate acceptance among animal health practitioners and policymakers [104].

Third, aligning veterinary, food safety, and public health policies is essential to addressing zoonotic tuberculosis along the entire farm-to-fork continuum. This alignment should include harmonized case definitions for bovine and zoonotic tuberculosis, coordination between meat and dairy inspection systems and livestock surveillance data, and the incorporation of *Mycobacterium bovis* differentiation into human tuberculosis guidelines in endemic settings. Regulatory frameworks ought to explicitly recognize unpasteurized dairy products and informal markets as critical control points and promote context-appropriate measures such as pasteurization, traceability, and certification schemes that are feasible for small-scale producers [105].

Key stakeholders include national veterinary services, ministries of health, food safety agencies, laboratory networks, producer organizations, and academic institutions, many of which are already involved in bovine tuberculosis control but often operate in relative isolation. Building existing initiatives such as national eradication programs and international roadmaps for bovine and zoonotic tuberculosis could provide a governance structure for joint planning and evaluation. The limited implementation of previous recommendations by international agencies reflects not only resource constraints but also the absence of mechanisms that translate technical guidance into binding national policies. Future efforts should therefore prioritize measurable indicators of One Health integration, including the proportion of human tuberculosis cases with species-level diagnosis, the number of outbreaks investigated jointly by veterinary and public health teams, and the coverage of molecular surveillance in high-risk livestock and dairy systems [106].

## 10. Conclusions

Bovine tuberculosis remains a persistent zoonotic challenge at the intersection of animal health, food safety, and public health, particularly in endemic regions such as Mexico and Latin America. Despite decades of control efforts, the continued circulation of *Mycobacterium bovis* in livestock, wildlife reservoirs, and human populations demonstrates that conventional eradication strategies, when applied in isolation, are insufficient to interrupt complex transmission networks.

This review highlights that the persistence of bovine tuberculosis is driven not only by biological factors but also by structural limitations in surveillance systems. Molecular epidemiology has unequivocally shown that *M. bovis* transmission occurs across interconnected animal, food, and human interfaces, yet these insights are rarely translated into routine control programs. Tools such as spoligotyping, MIRU-VNTR, and whole-genome sequencing have provided compelling evidence of zoonotic transmission and regional strain structuring, underscoring the need for surveillance strategies that move beyond aggregated prevalence estimates toward transmission-informed interventions.

From a diagnostic perspective, no single method offers adequate performance across all epidemiological contexts. Reliance on standalone screening tools can lead to underdetection and false confidence in disease status. Instead, integrated diagnostic algorithms—combining field-applicable immunological tests with targeted molecular confirmation—represent the most robust approach for improving detection accuracy and supporting effective control measures in endemic settings.

The One Health framework emerges not as a theoretical concept but as a practical necessity. Fragmentation between veterinary services, food safety authorities, and public health systems continues to obscure zoonotic transmission pathways, delay appropriate diagnosis in humans, and limit the effectiveness of prevention strategies. Operational integration—through shared data systems, coordinated outbreak investigations, and routine differentiation of *M. bovis* in clinical and veterinary laboratories—is essential for translating scientific advances into measurable public health impact.

Ultimately, sustainable control of bovine tuberculosis will depend on the capacity to align molecular surveillance, diagnostic strategies, and intersectoral governance within regionally adapted One Health programs. In endemic regions, strengthening this integration is critical not only to reduce economic losses in livestock production but also to mitigate the often-overlooked burden of zoonotic tuberculosis in human populations. Bridging the gap between available scientific tools and their practical implementation represents the most urgent priority for advancing toward the long-term control and eventual elimination of this neglected zoonosis. From an operational perspective, integrating whole-genome sequencing into national programs could be facilitated through phased approaches that leverage existing reference laboratories, public–private partnerships with the dairy and meat industries, and, where feasible, mobile or regional sequencing units to support high-risk areas.

## Figures and Tables

**Table 1:** Comparative overview of molecular epidemiology tools used for *Mycobacterium bovis* surveillance.

Method	Epidemiological Resolution	Transmission Tracing Capacity	Main Applications	Key Limitations
Spoligotyping	Low to moderate	Regional clustering and lineage identification	Baseline molecular surveillance Identification of dominant genotypes	Limited discriminatory power Inability to resolve recent transmission events
MIRU-VNTR	Moderate to high	Local outbreak investigation and herd-level comparisons	Refinement of transmission networks Differentiation within dominant spoligotypes	Locus variability Reduced comparability across laboratories
Whole-genome sequencing (WGS)	Very high	Direct transmission inference based on SNP differences	High-resolution phylogenetics One Health surveillance linking animal, food, and human cases	Higher cost Infrastructure and bioinformatics requirements

## Data Availability

No new data were created or analyzed in this study. Data sharing is not applicable to this article.

## Abbreviations

The following abbreviations are used in this manuscript:

bTB	Bovine Tuberculosis
MTBC	Mycobacterium Tuberculosis Complex
*M. bovis*	*Mycobacterium bovis*
*M. tuberculosis*	*Mycobacterium tuberculosis*
WGS	Whole-Genome Sequencing
MIRU-VNTR	Mycobacterial Interspersed Repetitive Units–Variable Number Tandem Repeat
SNP	Single-Nucleotide Polymorphism
PPD	Purified Protein Derivative
WHO	World Health Organization
WOAH	World Organization for Animal Health
FAO	Food And Agriculture Organization
PRONABIVE	Programa Nacional de Biológicos Veterinarios
SENASICA	Servicio Nacional de Sanidad, Inocuidad y Calidad Agroalimentaria
ELISA	Enzyme-Linked Immunosorbent Assay
IGRA	Interferon-Gamma Release Assay
